# Designing a privacy-preserving protocol to support transient and purpose-specific data linkages

**DOI:** 10.23889/ijpds.v1i1.374

**Published:** 2017-04-19

**Authors:** Chris Smith, Jenny Hewison, Allan House

**Affiliations:** 1 University of Leeds

## Objectives

To describe the design of a privacy-preserving protocol to support transient and purpose-specific data linkages between defined organisations in the health domain, to discuss how the specific technical and governance constraints of each organisation shaped the design of the protocol, and to propose approaches to reduce the costs associated with the use of privacy-preserving protocols for such linkages.

## Approach

Privacy-preserving protocols enable individual-level data to be linked across organisations without any requirement for direct identifiers to be released outside of organisational boundaries. Such protocols can reduce the risk that data released by organisations can be associated with an identifiable subject. Use of these protocols in practical scenarios requires that their computational and communication steps are mutually acceptable to each participating organisation from a technical and governance perspective. For transient and purpose-specific linkages, organisations may have no previous experience of participation in a privacy-preserving protocol and therefore no established set of mutually acceptable steps. We describe the design of a privacy-preserving protocol for a national health research project - in which specific health data from local and national organisations is to be linked to construct longitudinal patient records for analysis - and discuss how the design of the protocol was shaped by the specific technical and governance constraints of each organisation.

## Results

Organisations are subject to varied technical constraints, such as expertise and infrastructure, and varied governance constraints, such as policies and procedures. Therefore, different computational and communication steps may be acceptable to different organisations. Design of a privacy-preserving protocol requires interaction with each organisation to determine their specific constraints and to understand the space of mutually acceptable steps from which a protocol can be composed. Initial costs for the design of a privacy-preserving protocol can therefore be significant. However, once mutually acceptable steps have been established between certain organisations, ongoing costs for subsequent linkages should be reduced. To reduce both initial and ongoing costs, different approaches can used to support coordination of organisations on technical and governance matters. Such approaches could include: (i) education and training, (ii) tooling and accreditation, and (iii) published best practice.

## Conclusion

Use of privacy-preserving protocols for transient and purpose-specific data linkages requires coordination between organisations, which can be initially costly to establish. Reduction in initial and ongoing costs can be achieved through approaches that support both initial and ongoing coordination.

